# Food-Dependent Exercise-Induced Anaphylaxis: A Distinct Form of Food Allergy—An Updated Review of Diagnostic Approaches and Treatments

**DOI:** 10.3390/foods12203768

**Published:** 2023-10-13

**Authors:** Witchaya Srisuwatchari, Kantima Kanchanaphoomi, Jutamard Nawiboonwong, Torpong Thongngarm, Mongkhon Sompornrattanaphan

**Affiliations:** 1 Division of Allergy and Immunology, Department of Pediatrics, Faculty of Medicine, Siriraj Hospital, Mahidol University, Bangkok 10700, Thailand; witchaya.sr@gmail.com (W.S.); iewkantima@gmail.com (K.K.); 2 Faculty of Medicine, Center of Research Excellence in Allergy and Immunology, Siriraj Hospital, Mahidol University, Bangkok 10700, Thailand; j.nawib@gmail.com (J.N.); torallergy@gmail.com (T.T.); 3 Department of Medicine, Faculty of Medicine, Khon Kaen University, Khon Kaen 40002, Thailand; 4 Division of Allergy and Clinical Immunology, Department of Medicine, Faculty of Medicine, Siriraj Hospital, Mahidol University, Bangkok 10700, Thailand

**Keywords:** anaphylaxis, challenge test, food allergy, food-dependent exercise-induced anaphylaxis, gluten, IgE, oral food challenge, provocation test

## Abstract

Food-dependent exercise-induced allergic reactions (FDEIA) represent a distinct clinical phenomenon where symptoms arise during exercise following the consumption of specific trigger foods, with the most severe manifestation being anaphylaxis—a condition distinct from typical exercise-induced or food-induced anaphylaxis. In FDEIA, patients can either exercise or tolerate specific foods separately without experiencing any allergic reactions. Diagnosis relies on patient history and provocation testing, requiring rigorous implementation within a supervised hospital environment. Positive symptoms and clinical signs during testing confirm FDEIA, while negative outcomes do not preclude its presence. Exercise stands as the primary trigger, followed by nonsteroidal anti-inflammatory drugs (NSAIDs) and alcohol. The utilization of various protocols for food cofactor challenges to confirm FDEIA yields differing diagnostic outcomes. We highlight the updated concept of food cofactor challenges, incorporating protocols reported in the literature, and summarize current recommendations and comprehensive management approaches for FDEIA patients.

## 1. Introduction

Food-dependent exercise-induced allergic reactions (FDEIA) represent a unique clinical phenomenon. In FDEIA, reactions manifest when exercise or other cofactors coincide with the consumption of specific trigger foods, with the most severe manifestation being anaphylaxis [1,2]. Anaphylaxis represents the most severe end of the spectrum of allergic reactions, characterized by typical cutaneous symptoms (urticaria and/or angioedema) and associated with at least one other organ system. These systems can include respiratory symptoms (e.g., dyspnea, wheezing, bronchospasm, stridor, reduced peak expiratory flow, hypoxemia), cardiovascular symptoms (e.g., hypotension, collapse, syncope, incontinence), or severe gastrointestinal symptoms (e.g., severe crampy abdominal pain, repetitive vomiting). Anaphylaxis can occur following exposure to a known or probable allergen for that patient and may involve respiratory and/or cardiovascular compromise [3].

FDEIA is distinct from both typical exercise-induced anaphylaxis (EIA) and food-induced anaphylaxis [3,4,5]. In FDEIA, the interplay between food intake and physical exertion is essential for triggering anaphylactic reactions. Nevertheless, a comprehensive understanding of the intricate relationship between reaction timing, trigger factors, implicated foods, type of exercise, and cofactors, such as nonsteroidal anti-inflammatory drugs (NSAIDs) and alcohol, is lacking [2,6,7,8,9]. Age at the onset of FDEIA can vary, and they sometimes begin in childhood and adolescence, but onset most commonly occurs during adulthood [10,11,12]. Wheat and crustaceans are the most common trigger foods [5,12]. Exercise serves as the foremost trigger for FDEIA, followed by NSAIDs and alcohol [1]. The intensity and duration of exercise, ranging from vigorous workouts to routine activities, can provoke FDEIA symptoms, and there is a notable correlation between heightened exertion and an increased risk of allergic reactions [2,12]. Interestingly, documented cases of anaphylaxis occurring at rest add complexity to the FDEIA phenomenon [7]. Diagnosing FDEIA relies on a combination of patient history and allergic and provocation testing, both of which require rigorous implementation within a supervised hospital setting. While positive symptoms and clinical signs during testing confirm the presence of FDEIA, it is crucial to note that negative results do not rule out the possibility of this condition [11,13].

## 2. The Importance of Cofactors

In the domain of food allergies, FDEIA stands out as a distinctive clinical phenotype in which cofactors play a pivotal role, serving as essential triggers for allergic reactions [4,14]. Moreover, these cofactors, whether individually or in combination, can both lower the threshold for the causative food and exacerbate the severity of the allergic response [7]. Among the well-described cofactors, exercise takes precedence, followed by NSAIDs (e.g., aspirin, ASA) and alcohol [15]. Less common precipitating factors encompass physical stress, infection, menstruation, high temperature, and humidity [1,2,12,16,17].

A study from Brockow et al. [18] revealed that in 75% of wheat-dependent exercise-induced anaphylaxis (WDEIA) cases, specific cofactors—namely exercise, 500–1000 mg of ASA, and/or 10 to 30 mL of alcohol—were required to induce reactions. These findings were corroborated by Christensen et al. [15], who reported similar results. In the case of 25 adult patients diagnosed with WDEIA, the presence of additional cofactors led to a significant reduction in the clinical reaction threshold. Specifically, when considering exercise alone, the threshold for food tolerance decreased by 63%. With the inclusion of aspirin, this reduction increased to 83%, and when alcohol was added, it decreased by 36%. In the presence of both exercise and aspirin, the reduction reached 87% compared to reactions that occurred during rest [15].

Symptoms of FDEIA can manifest in response to both strenuous and milder forms of exercise, with some patients even experiencing reactions during routine physical activities like walking or climbing stairs. While higher exercise intensity and longer duration increase the likelihood of symptom triggering, instances of anaphylaxis at rest have also been documented [19]. A study conducted by Christensen et al. [7] demonstrated that 26 out of 71 patients (37%) with WDEIA could experience reactions even at rest. These reactions, however, were less severe, and the patients exhibited a higher tolerance for wheat protein compared to those patients who experienced reactions during exercise. However, exercise does not appear to be an obligatory trigger, as WDEIA can be confirmed in a challenge setting even at rest (without the presence of cofactors), as long as the implicated food (in this case, gluten) is ingested in a sufficiently substantial quantity. Consequently, exercise can be regarded as an augmenting factor capable of lowering the threshold and increasing the severity of reactions.

**Figure 1:**
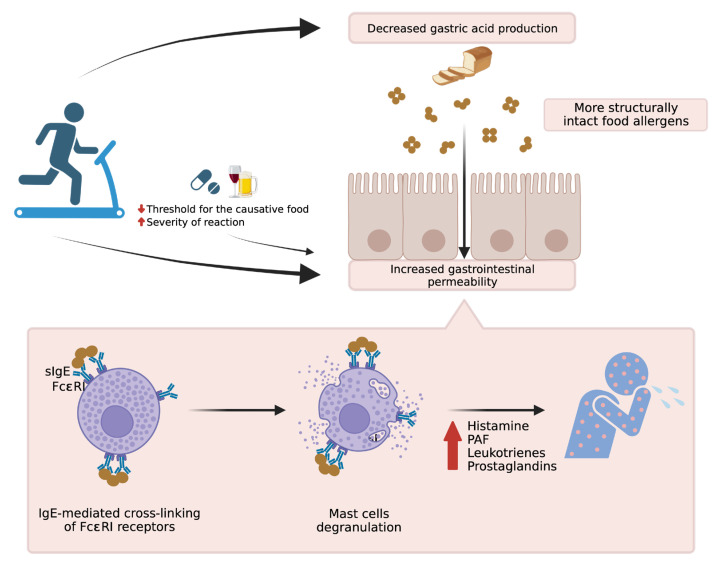
Pathophysiology of food-dependent exercise-induced anaphylaxis (FDEIA). Created via BioRender.com (accessed on 2 October 2023).

In addition, ASA has been reported to exacerbate FDEIA symptoms when taken in conjunction with the triggering food, regardless of whether exercise is involved. Motomura et al. [8] revealed that among children with FDEIA, incorporating ASA pretreatment during confirmed food and exercise challenge testing led to an approximate 50% increase in positive reactions compared to tests without ASA pretreatment. The pathomechanisms underlying FDEIA are briefly summarized in Figure 1.

Physical activity enhances gastrointestinal permeability while reducing gastric acid production, leading to increased absorption of structurally intact nutrients. The sizeable molecules from undigested foods bind to specific IgE antibodies on mast cells, subsequently triggering mast cell activation through IgE-mediated cross-linking of FcεRI receptors. This activation, in turn, prompts the release and de novo synthesis of inflammatory mediators and cytokines associated with anaphylaxis. Additionally, cofactors such as nonsteroidal anti-inflammatory drugs (NSAIDs) or alcohol have demonstrated the ability to heighten gastric permeability and modify tight junctions within the gastrointestinal tract epithelium. These alterations contribute to a lowered threshold for adverse food reactions or exacerbate allergic reaction severity.

## 3. Atopic Diseases and Related Comorbidities of FDEIA

A recent systematic review of FDEIA [2] patients reported that 59.7% of patients with FDEIA had coexisting atopic conditions. Among these, the most prevalent atopic comorbidities included allergic rhinitis, asthma, allergic conjunctivitis, and atopic dermatitis. It is worth highlighting that certain atopic diseases, such as asthma, may exhibit increased severity during episodes of FDEIA. Consequently, it is imperative to thoroughly evaluate patients for concurrent atopic conditions and ensure they receive appropriate management and care tailored to their specific needs. Furthermore, it is important to consider that a history of atopy may potentially act as a significant risk factor for the occurrence of recurrent episodes. A recent systematic review [2] demonstrated that in the univariate analysis, various factors such as gender (female), a history of atopy (specifically allergic rhinitis), and the identification of wheat as the causative food were all significantly associated with recurrent episodes of the condition. However, upon conducting a multivariate analysis, it became evident that the history of atopy, particularly allergic rhinitis, remained the singular and significant risk factor for such recurrences.

In addition, it is worth noting that 37.2% of individuals reported a history of urticaria, with chronic spontaneous urticaria being the most frequently documented subtype [2]. The link between food allergies and chronic urticaria has been commonly recognized as part of the broader framework of multiple morbidities often seen in atopic diseases. Intriguingly, both of these conditions involve mast cells as key players in their pathophysiology. While recent research has shed light on the presence of distinct mast cell subpopulations with varying functions, there is a compelling need to delve deeper into potential shared mechanisms or common pathways that may exist between these two conditions. Future investigations in this direction hold the promise of unveiling novel insights into their interplay and therapeutic opportunities [20].

Concomitant treatment for chronic urticaria has the potential to mask or delay the recognition of an IgE-mediated reaction triggered with various allergens, as it may partially conceal or delay the appearance of skin-related symptoms. In cases where there is an unexpected change in the severity or frequency of chronic urticarial symptoms that were previously well-controlled, or the onset of systemic symptoms, it is prudent to consider the possibility of an IgE-mediated reaction. Special attention should be given to patients who experience exacerbated urticaria during exercise, as this could be indicative of FDEIA. Keeping a detailed food diary that includes information about cofactors and their temporal relationship with symptoms can provide valuable clues for the diagnosis of FDEIA [21].

## 4. Diagnosis of FDEIA

### 4.1. Clinical Diagnosis

Diagnosing FDEIA is a complex task that relies on clinical evaluation, and it remains challenging on multiple fronts. Patients often experience recurrent episodes of reactions, leading to delayed and sometimes inaccurate diagnoses [1,4,19].

Traditionally, it has been understood that individuals with FDEIA can tolerate specific foods or augmenting factors independently, but reactions occur when exercise or other cofactors coincide within 4 h of food ingestion [5]. Interestingly, the reverse scenario, where food is consumed just after exercise, can also trigger reactions [12]. The clinical manifestations of FDEIA span a spectrum from mild cutaneous reactions (e.g., urticaria) to potentially life-threatening anaphylaxis [2]. Notably, anaphylactic responses in FDEIA tend to be more severe compared to typical IgE-mediated food-induced anaphylaxis. This heightened severity is often due to the frequent involvement of cardiovascular symptoms (e.g., hypotension, syncope, loss of consciousness), along with respiratory symptoms (e.g., chest tightness, wheezing, cough, desaturation) [11,12,13,22,23].

Furthermore, FDEIA is frequently misdiagnosed, as it shares clinical features with other conditions, including chronic or acute recurrent urticaria, exercise-induced anaphylaxis, idiopathic anaphylaxis, hereditary angioedema, and mastocytosis [3,24,25].

### 4.2. In Vivo and In Vitro Tests

In confirming the diagnosis of FDEIA, it is crucial to consider both the patient’s clinical history and the presence of positive specific IgE (sIgE) and/or a positive skin prick test (SPT) related to the suspected food [1,19]. While wheat has emerged as the most commonly reported trigger for FDEIA, a wide range of other foods have also been implicated, including seafood, red meat, eggs, buckwheat, peanuts, tree nuts, fruits, and vegetables [12,16,17,19,26,27].

In cases where allergen extracts or sIgE for the suspected foods are not commercially available, a prick-to-prick test (PTP) can be conducted. It is important to note that a positive outcome in either in vivo or in vitro tests does not always definitively confirm an allergy. Therefore, the selection of tests should be based on clinical history and limited to the relevant causative food. In cases where a suspected allergen cannot be identified or does not align with the clinical history, the consideration of an exercise–food challenge test becomes necessary to achieve an accurate diagnosis [5].

In the context of WDEIA, a significant allergen component known as omega-5-gliadin (ω5-gliadin) has been identified—initially observed in 18 Finnish adults with WDEIA, all exhibiting IgE antibodies to this novel ω5-gliadin [28]. Matsuo H, et al. [29] demonstrated an 80% sensitivity in 50 Japanese children and adults with WDEIA using sIgE to ω5-gliadin (with a cut-off value > 0.35 kUA/L) determined via ImmunoCAP^TM^ (Phadia, Uppsala, Sweden). From the ROC analysis, an optimal cut-off value for ω5-gliadin sIgE was determined to be 0.89 kUA/L. This value, which yielded a sensitivity of 78% and a specificity of 96%, was recommended for use [29]. A study from Brockow K, et al. [18] produced similar findings among 16 adults with challenge-confirmed WDEIA. The sensitivities of sIgE to wheat, gluten, and ω5-gliadin were reported as 81%, 100%, and 100%, respectively, with corresponding specificities of 87%, 95%, and 97%, using a cut-off value > 0.35 kUA/L. Additionally, the PTP employing gluten demonstrated a sensitivity of 100% and a specificity of 96%.

In cases where patients manifest multiple episodes of severe allergic reactions or anaphylaxis and are suspected of having FDEIA, it is advisable to measure basal serum tryptase levels. Elevated baseline values should then trigger an assessment for mastocytosis or other mast cell disorders [1].

### 4.3. Food–Exercise Cofactor Challenge Test

The exercise challenge test, conducted subsequent to the oral food challenge test (OFC), henceforth referred to as the exercise–food challenge test, is a valuable diagnostic tool when a definitive diagnosis cannot rely solely on clinical history, in combination with SPT and/or sIgE. Despite being considered the gold standard test, the exercise–food challenge test’s principal limitation is its positivity rate, ranging from 50 to 100% across protocols [6,7,8,11,13,18,19,26,30]. This variability arises from complex interactions between factors like exercise type/duration/intensity, ambient room conditions, temperature, and humidity, collectively influencing reaction reproducibility. Therefore, the negative challenge test may indicate challenges in selecting the causative foods, identifying contributing factors, or ensuring the appropriate intensity and duration of exercise during testing. Although several protocols have been established, there is currently a lack of standardization or consensus, encompassing factors such as food protein challenge quantity, exercise duration/intensity, cofactor type/dosage, and protocol duration. Typically, the exercise–food challenge test begins with substantial food protein consumption, optionally supplemented with ASA (10 mg/kg/dose in children, 300–1000 mg in adults) and/or alcohol. This is followed by aerobic and/or anaerobic exercise, lasting 4–6 min or extending up to 60 min, initiated 30–60 min after food ingestion. Some authors recommend maintaining the challenge room at 25–30 °C with 40–50% humidity [7,11,13,18,19,26,30,31,32]. Table 1 summarizes the studies conducting food–exercise cofactor challenge tests in the previous literature.

OFCs, combined with exercise cofactors, serve as the established standard for confirming FDEIA diagnosis. However, when employing the combined wheat–exercise challenge, negative outcomes have been documented. As effective alternatives or supplements, ASA and alcohol can elicit symptoms, with ASA not only facilitating reactions but also lowering the challenge threshold. This suggests potential enhancement of diagnostic yield, particularly for cases where patients are unable to attain the target exercise intensity.

**Figure 2:**
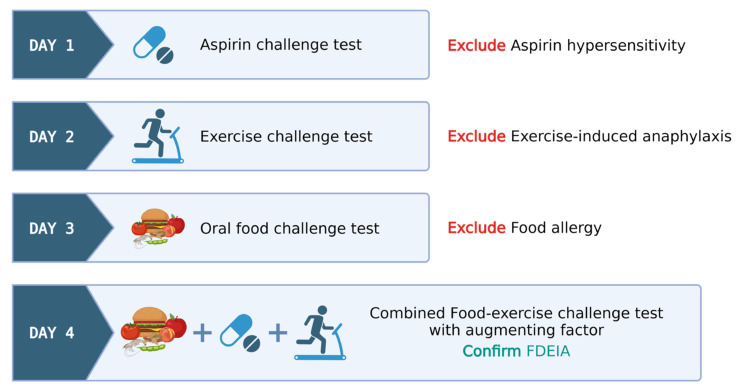
Illustrative model for food–exercise cofactor challenge test protocol in food-dependent exercise-induced anaphylaxis (FDEIA). Created via BioRender.com (accessed on 2 October 2023).

Protocols employing multiple cofactors have led to an increase in test positivity; in contrast, they are sometimes associated with more severe reactions. In such multi-cofactor protocols, it remains essential to confirm that patients do not exhibit allergic reactivity to these factors. Figure 2 exemplifies a sequential multi-day challenge test protocol, employing ASA and exercise in tandem after wheat ingestion, for diagnosing wheat-dependent exercise-induced anaphylaxis (WDEIA) or omega-5 gliadin syndrome.

**Day 1:** ASA provocation test, cumulative 421.5 mg, observed for 6 h to rule out ASA hypersensitivity.
**Day 2**: Exercise challenge at 27–30 °C ambient temperature. Treadmill exercise adjusted for target heart rate (>80% max HR) for 15 min. Test ceased at any positive reaction or gradually tapered if negative.
**Day 3**: Wheat challenge using common wheat source (Farmhouse^®^ bread, Thailand)—incremental dosing up to five slices observed for 6 h.
**Day 4**: Combined wheat–exercise–ASA challenge. ASA (300 mg) followed by four slices of bread, exercise as per Day 1.

The food–exercise challenge test protocols exhibit notable variability in their approaches. Nonetheless, these protocols consistently span several days and they are meticulously structured to encompass a broad range of scenarios, thereby precluding the influence of singular factors like exercise-induced anaphylaxis, food allergies, and hypersensitivity reactions to cofactors like aspirin. A favorable outcome in a combined food–exercise challenge test serves to conclusively establish the diagnosis of food-dependent exercise-induced anaphylaxis (FDEIA).

In cases where the exercise challenge yields a negative result on day 2, the patient proceeds to an open wheat challenge on day 3. Similarly, if a negative result occurs during the open wheat challenge on day 3, the patient advances to the combined wheat cofactor challenge on day 4. This protocol facilitates the diagnosis of EIA, classical IgE-mediated food allergy, and FDEIA on days 2, 3, and 4, respectively. If the physician chooses to exclusively employ exercise as the cofactor, the protocol may skip day 1, beginning directly with the exercise-only challenge.

Following the test, patients should undergo stringent monitoring for a minimum of 4 h, possibly extending to 24 h due to the possibility of late reactions [11]. It is important to emphasize that this test should be exclusively conducted under the supervision of a well-trained physician with adequate equipment and a trained personnel team, due to the risk of severe reactions [5].

## 5. Management of FDEIA

**Figure 3:**
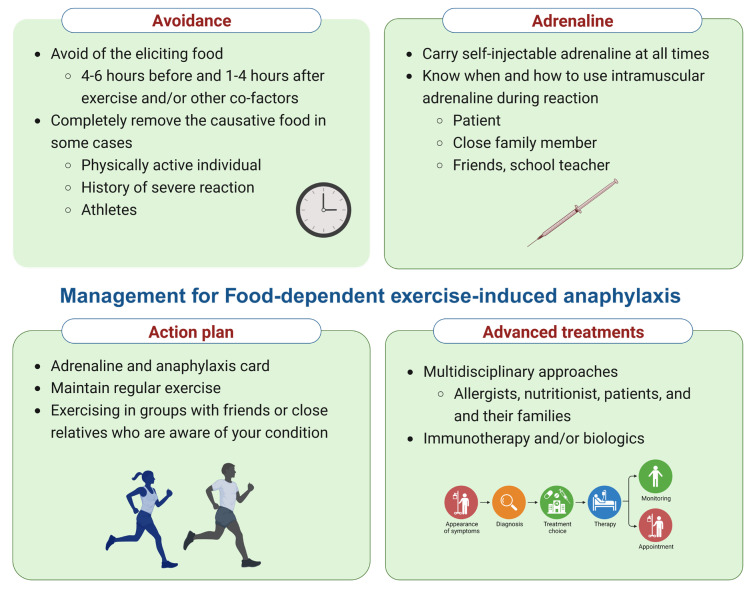
Management checklist for food-dependent exercise-induced anaphylaxis (FDEIA). Created via BioRender.com (accessed on 2 October 2023).

The management summary for FDEIA patients is shown in Figure 3 and Table 2. Prompt treatment of acute allergic reactions or anaphylaxis, particularly with epinephrine, is crucial [3,33,34]. Patients should have access to self-injectable adrenaline, receive education on anaphylaxis action plans and food labeling, and avoid the implicated food 4–6 h before and 1–4 h after exercise or other cofactors like ASA and alcohol. However, physically active patients with a history of severe reactions might need to completely avoid the causative food. Encouraging regular physical activities and carrying an anaphylaxis identification card at all times is essential [5]. Caution is advised for patients taking β-blocking agents and angiotensin-converting enzyme inhibitors, given their potential impact on anaphylaxis severity and epinephrine response.

There are currently no randomized controlled trials for preventing allergic reaction episodes with medications. Therefore, premedication with antihistamines, oral corticosteroids, and leukotriene receptor antagonists is not recommended. While immunotherapy and anti-IgE antibody treatments, like omalizumab, can be considered for certain patients with IgE-mediated food allergies, limited data exist for FDEIA, with only a few small case series reported [25].

## 6. Gaps in Knowledge and Future Directions

Significant knowledge gaps persist, posing fertile grounds for prospective research and clinical interventions. These gaps encompass the imperative to unravel the underlying pathomechanisms of FDEIA, particularly when symptoms manifest during periods of rest or routine physical activity, defying current explanatory models. Furthermore, a comprehensive understanding of the natural history and prognosis of FDEIA within a large cohort remains elusive. The principal impediment to such investigations lies in the absence of standardized methods for confirming natural tolerance. Given that FDEIA exhibits a relatively high threshold compared to classical IgE-mediated food allergies, it is imperative that studies on its natural history establish stringent criteria for defining “natural tolerance”.

Furthermore, there is another condition known as oral mite anaphylaxis that exhibits similarities to FDEIA, specifically referred to as dust-mite-ingestion-associated exercise-induced anaphylaxis. This condition is exceptionally rare, with only a limited number of documented case reports available to date. This underscores the noteworthy role of exercise as a contributing factor to anaphylactic reactions in individuals who have consumed mite-infested food. It appears that these reactions are triggered by heat-stable allergens, as our case and previous reports indicate that cooking the food does not significantly alter the outcomes [35].

Lastly, it is worth noting that, as of the present moment, there exists neither a defined treatment nor a preventive strategy for mitigating the development of FDEIA. This paucity of therapeutic and preventative measures poses a significant clinical challenge, as individuals afflicted with this condition often face recurrent episodes of severe allergic reactions, imposing a substantial burden on their quality of life and healthcare systems. Addressing this critical gap in our medical knowledge is paramount to improving patient care and outcomes in the context of FDEIA.

## 7. Conclusions

In conclusion, FDEIA presents a unique and complex clinical phenomenon that requires careful consideration of various factors, including food triggers, exercise intensity, cofactors like NSAIDs and alcohol, and the presence of atopic comorbidities. The diagnosis of FDEIA is challenging, often requiring a combination of patient history, allergic testing, and provocation tests conducted in a supervised hospital setting. The role of cofactors, particularly exercise, is pivotal in this condition, as it can lower the threshold and increase the severity of reactions.

Furthermore, the association between FDEIA and atopic conditions, such as allergic rhinitis and asthma, highlights the importance of comprehensive patient evaluation and tailored management strategies. Patients with a history of atopy, especially allergic rhinitis, might be at a higher risk of recurrent episodes. While progress has been made in understanding FDEIA, many knowledge gaps remain, including the elucidation of underlying pathomechanisms, natural history, and prognosis of the condition. Additionally, the absence of defined treatments or preventive strategies underscores the need for further research to address this clinical challenge and improve patient care and outcomes in the context of FDEIA.

## Figures and Tables

**Table 1:** Summary of studies conducting food and cofactor challenge tests in individuals suspected of food-dependent exercise-induced anaphylaxis (FDEIA).

Study, Country	Population	Protocol	Challenge Food	Exercise	Cofactors	Positive Challenge Rate
Studies in children and adolescents						
Motomura et al. [8] 2015, Japan	51 children	3 days	Noodles (70 g of wheat), boiled shrimp (50 g)	30 min of aerobic exercise followed by 6 min of anaerobic exercise	ASA 10 mg/kg/dose (Max 500 mg)	51%
Asauimi et al. [26] 2016, Japan	41 children and adolescents	1 day	Udon noodles (200–400 g of wheat, 5.2–10.4 g of wheat protein), shrimp (40–100 g), or one whole apple	Ergometer ≥ 6 min	ASA 10 mg/kg/dose (Max 500 mg)	49%
Studies in children, adolescents, and adults						
Aihara et al. [6] 2002, Japan	10 children, adolescents, and adults	2–5 days	Wheat (100 g of wheat)	No detail	ASA 500 mg	80%
Srisuwatchari et al., [11] 2021, Thailand	14 children, adolescents, and adults	3 days	Bread (60–75 g of wheat, 7.6–9.5 g of wheat protein)	Motor-driven treadmill Children and adolescents ≥ 4 min and adults ≥ 15 min	ASA Children 10 mg/kg/dose (Max 300 mg) Adults 300–381 mg	71.4%
Studies in adults						
Brockow et al. [18] 2015, Germany	16 adults	6 days	Baked bread (10–80 g of pure gluten flour)	45 min of aerobic exercise followed by 8 min of anaerobic exercise	ASA 500–1000 mg and 10–30 mL of 95% ethanol	100%
Christensen et al. [7] 2018 Denmark	71 adults	2 days	Baked bread (80 g of pure gluten flour)	4 treadmill aerobic phases of 15 min	None	62%
Christensen et al. [15] 2019, Denmark	25 adults	5 days	Baked gluten rolls (80 g)	Treadmill aerobic exercise for 15 min	ASA 1000 mg, 37.5% alcohol	100%
Thongngarm et al., [13] 2020, Thailand	18 adults	3 days	Bread (60–75 g of wheat, 7.6–9.5 g of wheat protein)	Motor-driven treadmill for 15 min	ASA 300 mg	94.4%

Abbreviation: ASA, acetyl salicylic acid; g, gram(s); kg, kilogram(s); mg, milligram(s); min, minute(s).

**Table 2:** Summary of management strategies for food-dependent exercise-induced anaphylaxis (FDEIA) patients.

Treatment of the acute episode of FDEIA	
Treatment for anaphylaxis	Assess airway, breathing, and circulation. Administer intramuscular injection of epinephrine (1:1000) at 0.01 mg/kg/dose (maximum: 0.5 mg for adults or 0.3 mg for children); consider repeating the dose in 5–15 min if necessary. Place the patient in a supine or a Trendelenburg position. Provide supplemental oxygen as needed. Initiate intravenous fluid therapy. Initiate cardiopulmonary resuscitation (CPR) in the event of cardiopulmonary arrest. Consider second-line medications: antihistamines, bronchodilators, and systemic corticosteroids. Monitor the patient for a minimum of 4–8 h to detect any potential late-phase reactions.
Patient education	
Anaphylaxis action plan	Recognize the signs and symptoms indicative of anaphylaxis. Understand the potential risk of future recurrent reactions. Possess and be proficient in using self-injectable epinephrine. Demonstrate competence in interpreting food labels.
Food avoidance	Refrain from consuming the triggering food for 4–6 h prior to exercise and within 1–4 h following exercise, as well as during exposure to other contributing factors such as aspirin or alcohol. For active children, adolescent athletes, and individuals engaged in physically demanding occupations with a history of severe reactions, complete elimination of the responsible food from their diet may be recommended.
Physical activity	Promote the continuation of regular exercise. Advocate for exercising in group settings within populated areas and emphasize the importance of carrying an anaphylaxis identification card at all times.
Underlying patient knowledge	Convey the need to take β-blockers and angiotensin-converting enzyme inhibitors with caution since they increase the severity of anaphylaxis and reduce the response of epinephrine
Prophylaxis and treatment	
Pharmacological prophylaxis	Not recommended to take any premedication to prevent the allergic reaction, e.g., antihistamine, oral corticosteroid, or leukotriene antagonist inhibitor
Biological agents	Absence of current recommendations
Desensitization of the causative food	Absence of current recommendations

## Data Availability

Not applicable.
